# Family Club Denmark: A Quasi-Randomized Study of a Volunteer-Based Intervention to Support Vulnerable Families

**DOI:** 10.3390/healthcare12111115

**Published:** 2024-05-29

**Authors:** Maiken Pontoppidan, Mette Thorsager, Arendse T. Larsen, Mette Friis-Hansen

**Affiliations:** VIVE—The Danish Centre for Social Science Research, Herluf Trolles Gade 11, 5200 Copenhagen, Denmark; meje@vive.dk (M.T.); artl@vive.dk (A.T.L.); mfh@vive.dk (M.F.-H.)

**Keywords:** volunteer, parenting, family, intervention, effectiveness

## Abstract

Volunteer interventions play a vital role in supporting families by offering accessible and community-based resources outside the formal professional sector. This study examines the impact of the volunteer intervention known as Family Club Denmark (FCD) on the well-being of parents and children. FCD aims to provide families with positive experiences and support relationship building. The intervention, open to families from diverse social backgrounds, comprises volunteer-led family clubs where parents and children aged 2–12 years engage in activities and meals. We allocated 510 families (363 vulnerable families) to FCD or placed them on a waiting list based on a first-come, first-served principle. We conducted baseline, post-intervention, and follow-up assessments through questionnaires, observations, and interviews. On average, families participated in 5.8 sessions, with both families and volunteers reporting high satisfaction. When compared to control families, we find that vulnerable FCD parents feel more confident playing with their children (*p* = 0.04, [0.01; 0.40], d = 0.25), require less assistance in playing with their children (*p* = 0.01, [−0.34; −0.05], d = 0.33), and report that their children have a more challenging time forming friendships (*p* = 0.01, [−0.51; −0.09], d = 0.29). However, we did not find significant effects on mental health, parenting stress, self-efficacy, self-worth, family routines, or child well-being. We observed similar results for the full sample. The discovery that parents feel more confident playing with their children after participating in FCD highlights the vital role of volunteer-based interventions in enhancing parental engagement and fostering positive parent–child interactions. Trial registration: ClinicalTrials.gov NCT03657888 (registered 29 August 2018).

## 1. Introduction

Within the last decades, civil society in the Nordic countries has changed. Today, civil society provides services that contribute to the well-being and quality of life of volunteers and participants and is increasingly seen as a welfare state problem solver [1,2]. This has resulted in a stronger interest in developing collaboration between the voluntary sector and municipalities and a stronger focus on price and cost-effectiveness [2]. Because voluntary activities within civil society belong to neither the private nor the public sectors, they are often referred to as ‘the third sector’. 

In Europe, the third sector mobilizes a volunteer workforce corresponding to over 15.5 million full-time equivalent workers, indicating the vast reach and power of the third sector [3]. Volunteers usually receive some job-related training but have no formal professional education in their volunteer field. Due to their democratic structures and innovative solutions to problems, the activities of the third sector increasingly contribute to solving complex problems such as social problems, loneliness, and health problems [4,5,6,7]. Therefore, voluntary services are considered valuable, especially when costly professional services are shifted towards non-professional, inexpensive support that volunteers offer. 

Family Club Denmark (FCD) is a voluntary intervention where families with different social backgrounds meet, play, and eat together every second week for six months. FCD is a family support intervention aiming to increase the network for children and parents in vulnerable families, promote positive parenting skills, and improve the well-being of parents and children.

### Effect Studies of Volunteer Family Support Interventions

High-quality evidence of the effects of voluntary interventions within health and social care is scarce [8]. Efficacy trials require strict control of different aspects of a trial, such as strict inclusion criteria, training and background of clinicians, and a manual-based intervention. Studies of voluntary interventions have a hard time meeting these criteria, mainly because of the inherently unique nature of voluntary social work. Volunteers are not paid employees who act according to the expectations of the administrative system. On the contrary, volunteers are guided by interactional logic, driven by situational devotion, and act based on meaningful relationships [9]. Therefore, studies of voluntary interventions are often conducted as pragmatic trials. 

Pragmatic trials are designed to examine the effects of interventions in real-life settings and are, therefore, of great interest to decision makers [10]. Studies of volunteer interventions aimed at children and parents have been carried out in different areas such as left-behind children [11], reading interventions [12,13], mental health [14,15,16], and family support interventions [17,18,19,20,21,22,23,24,25,26,27,28,29,30]. 

Most studies of family support programs are conducted in low- and middle-income countries (LMICs). Two examples are the cluster-randomized pilot study of Rwanda’s cash-for-work Vision Umurenge [26] and a pilot trial of family therapy in Kenya [31,32]. On a meta-analytical level, three reviews of psychological interventions primarily based on studies from LMICs found promising results for mental health outcomes [33,34,35]. However, the researchers also call for more rigorous research [35]. 

Studies of the effects of volunteer family support programs in developed countries find mixed results. Kelleher and Johnson found that the Cottage Community Care Pilot Project aimed at vulnerable parents improved access to social support and age-appropriate infant expectations [27]. Chacko et al. examined the parenting intervention Caring in Chaos in a wait list RCT, including 161 parents of children with ADHD symptoms. They found positive effects of the intervention in parenting, parental stress and depression, and child functional impairment [30] and that the intervention was cost-effective compared to programs delivered by professionals [36]. Gardner et al. examined a video-based parenting intervention in an RCT, including 76 children aged 2–9 with conduct problems. They found significant improvements in child negative behavior, child independent play, and parenting but found no change in maternal depression [25]. 

Another example of a volunteer family support program is Home-Start, which since 1973 has offered volunteer support to vulnerable families with young children in many countries [37]. Evaluations of the Home-Start initiative find mixed results. One study could not conduct a quantitative analysis due to the poor quality of administrative data [4]. Two studies found some effects on parenting but no effects on child behavior [29,38]. Two studies did not find effects on maternal well-being [28,37]. Two studies with longer follow ups showed improvements in parental well-being, behavior, and competence during the intervention, and these changes were sustained until ten years after the intervention [19,21]. 

In addition to the inconsistent results found in both individual trials and in meta-analyses, many studies are challenged by methodological shortcomings such as high and potentially selective attrition rates [19,21,30,39,40,41], small sample sizes [19,21,25,26,27,28,29,38], lack of randomization [19,21,29,37,38], no comparison group [4], and no complete blinding of the observer [29,30]. 

In sum, methodologically sound studies on the effects of volunteer family support programs are limited, and findings are inconsistent due to differences in study methodologies, characteristics of the programs investigated, outcomes examined, and whether the study is conducted in LMICs. Despite inconsistent results, family support programs carried out by volunteers are still continuously being developed and implemented all over the world. This study examines the effects of the volunteer intervention of Family Club Denmark (FCD) on the well-being of parents and children. We also examine volunteers’ and families’ experience participating in FCD. 

## 2. Materials and Methods

This study is a prospective, pragmatic, quasi-experimental trial with two conditions: an intervention group participating in FCD and a wait list control group. We recruited families in all five Danish regions from September 2018 to December 2020. We conducted this study according to CONSORT guidelines [42,43]. More details about this study can be found in the study protocol [44]. 

### 2.1. Participants and Procedures

To participate, families had to meet the following inclusion criteria: (1) a desire to participate in FCD, (2) at least one child aged 2–12 years, and (3) the ability to fill out questionnaires in Danish. Families unable to complete questionnaires in Danish were excluded. The three partner organizations employed project coordinators to find venues, recruit volunteers and families, manage sign-ups on the website, and support volunteers. Most participants signed up through the FCD website, while some were recruited directly at local sites. Participants received written information about the project and provided electronic, written consent to participate in the trial.

The typical recruitment process was as follows. (1) Families applied for a specific family club through the website and consented to be contacted by VIVE. (2) The project coordinator compiled a list of participants. (3) The project coordinator allocated participants to either the intervention group or the wait list control group based on a first-come, first-served principle. (4) The project coordinator sent contact information and allocation details to VIVE. (5) The project coordinator informed VIVE about the starting dates of the family clubs. (6) VIVE distributed the baseline questionnaire, including electronic consent to participate via text message and email. (7) The project coordinator informed participants about their allocation. 

Families were also assigned to the wait list control group if there was no nearby FCD, if an FCD group was delayed by more than four months, if they had scheduling conflicts, or if they did not show up for FCD sessions.

### 2.2. Intervention 

The FCD concept was developed in 2017 in collaboration between three third-sector partner organizations. The principles are described in a concept book and a practical guide. Theoretically, FCD is based on social learning theory, neuroscience, and positive psychology. The intervention aims to provide families with positive experiences and support relationship building. Participating in FCD is not limited to vulnerable families, as the mix of social backgrounds and circumstances is one of the anticipated strengths of the intervention. Each family club has a volunteer team comprising a leader and two to five more volunteers. The volunteers are instructed to use praise and show each family that they are essential contributors to the family club. The volunteers are also trained to apply predictability and routines in the family club to create a program for each session, including the following elements: welcome, activity, dinner, and goodbye. 

FCD is based on four core principles: (1) Meal Community, (2) Play, Learning, and Togetherness, (3) Support and Advice, and (4) Bridging to Civil Society and the Public Sector. Each session must incorporate the first two principles. Volunteers attend one-day training sessions every six months, which are based on these principles and seven value posters. The training provides volunteers with guidance on body language, teamwork, and meeting facilitation. Experienced volunteers receive advanced training and team support. All volunteers are provided with the FCD concept book and a guidebook containing practical information on teamwork, fundraising, confidentiality, and insurance.

Each club consists of up to nine families who meet biweekly for six months, totaling twelve sessions. Participants have the option to continue for an additional six months upon completing the initial period. The twelve sessions are structured around seven value posters: (1) Fun with Smiles, (2) Together but Not in Line, (3) The Time is Now, (4) Notice and Say Thank You, (5) More than Me, (6) Courage to Dare, and (7) Taste the World. The FCD concept book elaborates on these seven values and the activities associated with them. 

### 2.3. Measures Families

We collected questionnaire data from parents through a secure online survey database at three time points: the baseline (T1), post-intervention (six months after the baseline—T2), and follow up (12 months after the baseline—T3). Participants received an email with a direct link to the questionnaire, and reminders were sent via text message and email. If families needed assistance with completing the questionnaire, they were contacted by a research assistant. As an incentive, families received a small gift at each of the three data collection points: a children’s cookbook at baseline, a 150 DKK (~20 EUR) electronic gift card at post-intervention, and a 100 DKK (~15 EUR) electronic gift card at follow up.

Socio-demographic characteristics include the parent’s age, education, occupation, ethnicity, number of children, child age, household status, housing situation, and household economy. Table 1 shows the timing of the administration of measures. More details about the measures are described in the study protocol [44].

#### Primary and Secondary Outcomes

This study’s primary outcome is the seven-item Short Warwick–Edinburgh Mental Well-being Scale (SWEMWBS) measuring well-being in adults [45]. A total score is calculated by summing the seven items and converting the raw score according to a published conversion table. Scores range from 7 to 35 for both raw and converted scores. A high score reflects a better outcome [46]. Cronbach’s alpha is 0.85.

The secondary outcomes include the following measures. 

Parent Behavior Inventory (PBI) is a twenty-item measure of parenting behavior with two subscales: supportive/engaged and hostile. We used a version with ten items. The score range is 0–25 for each subscale. A high score is better for supportive/engaged, and a low score is better for hostile/coercive [47]. Cronbach’s alpha is 0.62 for the supportive/engaged subscale and 0.55 for the hostile/coercive subscale. 

The Parental Stress Scale (PSS) is an eighteen-item measure of parenting stress consisting of two subscales: Parental Stress (PS) and Lack of Parental Satisfaction (LPS). Following [48], items were dichotomized (0–1), and items 2 and 11 were left out. However, items 3 and 4 were also left out due to a technical error. Total scores range from 0 to 7 (PS seven items) and 0 to 7 (LPS seven items reversed), and a low score is better [49]. Cronbach’s alpha is 0.78 for PS and 0.82 for LPS. 

The general self-efficacy scale (GSE) is a 10-item measure of optimistic self-beliefs to cope with challenging life demands [50]. We used a three-item version. The score range is 3–12, and a high score is better. Cronbach’s alpha is 0.86. 

Self-worth: We used three items from the HBSC project to measure self-worth. The score range is 3–15, and a high score is better [51]. Cronbach’s alpha is 0.86. 

Family routines: We used five items from the Child Routine Inventory (CRI—39-item version) and added five items on dinner routines and language [52]. The score range is 0–40, and a high score is better. Cronbach’s alpha is 0.68. 

KIDSCREEN-10 is a 10-item measure of child well-being used with children aged eight or older. The score range is 10–50, and a higher score is better [53]. For children aged seven or younger, we used items about child well-being from the questionnaire for 2–6-year-old children in the project BørnUngeLiv (boernungeliv.dk). Cronbach’s alpha is 0.63. Due to a technical error, the questions on child well-being were assessed later for many families.

### 2.4. Volunteers

The volunteers were asked to fill out two questionnaires: first, immediately after the training session, and second, around four months after training. The first questionnaire assessed satisfaction with the training session and the second assessed gender, age, family status, education, employment, motivation for becoming a volunteer, and collaboration with the project coordinators, with the volunteer group, and with other organizations. The volunteers were also asked about the characteristics of the family club such as location, number of sessions, and activities. One of the partner organizations interviewed the volunteer leaders about the number of participants and the number of sessions held at FCD.

### 2.5. Statistical Analysis

We include data from families who have responded to at least two questionnaires (baseline, six-month follow up, or 12-month follow up). Categorical data are presented as numbers and percentages, and continuous data are presented as means and standard deviations. Baseline tests for imbalances include the Chi-2 test for categorical data and the *t*-test for continuous measures or ordinal scale variables (*p* < 0.05). 

We perform an attrition analysis based on families with a complete baseline questionnaire to check for systematic dropout from this study by regressing an indicator of attrition from the baseline to the six-month follow-up survey on various baseline measures. A total of 610 families responded to at least one questionnaire. However, imputation of data is not feasible for the full sample, given a relatively high rate of missing observations, especially at T3, where we have 336 responses. However, to test the sensitivity of the results to missing data, using the sample with at least one questionnaire response at the baseline or T2, we impute missing variables with chained multiple imputations of missing variables and perform an effectiveness analysis on imputed datasets. 

The primary analysis is conducted post-intervention (T2) for the vulnerable sample. For each outcome, we test if the FCD group scores differently from the wait list control group using linear regression of the outcome on an intervention indicator with robust standard errors. The estimation model includes imbalanced baseline measures (*p* < 0.1) as control variables and year-specific fixed effects to capture changes due to COVID-19. The analyses are repeated for the full sample post-intervention (T2). We applied two-sided tests with 0.05 significance levels throughout. We could not conduct an effect analysis for the second time period from T2 to T3 because both intervention and control families could participate in FCD in this time period. We, therefore, examine outcomes at follow up (T3) for the FCD group to only examine persistence in treatment effects or additional effects from a second round of FCD. 

We conduct subgroup analyses as stated in the protocol paper to examine potential differences between the following subsets of participants: vulnerability (vulnerable or non-vulnerable families); family composition (single parents or cohabiting parents); the age of the target child (<eight years old or ≥eight years old); and the number of sessions attended (dose). We also added a subgroup analysis where we examined the impact of COVID-19 on the effects of FCD. Due to a lack of data, we could not conduct subgroup analyses concerning partner organizations or the number of volunteers in FCD. 

### 2.6. Sample Size Justification

We recruited a larger sample than the planned 250 families in the full sample and 200 vulnerable families. With a total sample of 399 participants post-intervention (240 FCD and 159 control), we could detect a standardized mean difference (SMD) of 0.29 with a type 1 error rate of 0.05 and power at 80%. This corresponds to an SMD = 0.34 for the at-risk subsample (288 participants—177 FCD and 111 control). 

### 2.7. Observations and Interviews

We observed and interviewed families, children, and volunteers in 14 family clubs in the period 2018–2019 and interviewed 15 families in 2020. The 14 family clubs we visited varied with respect to characteristics such as rural/urban location, partner organization, and single parents/families to make sure that we gain in-depth knowledge of the variation in FCD and how the family clubs are implemented in different settings. The 2020 interviews were conducted by phone due to COVID-19 restrictions. The interviews focused on motivations for and experiences of participating in the family clubs and were centered on the following themes: breathing space, motivation, networking, and ending the family club. Finally, we analyzed individual phone interviews with 91 volunteer leaders conducted by one of the partner organizations. We recorded the interviews, transcribed them, and analyzed them using thematic analysis [54]. 

## 3. Results

**Figure 1:**
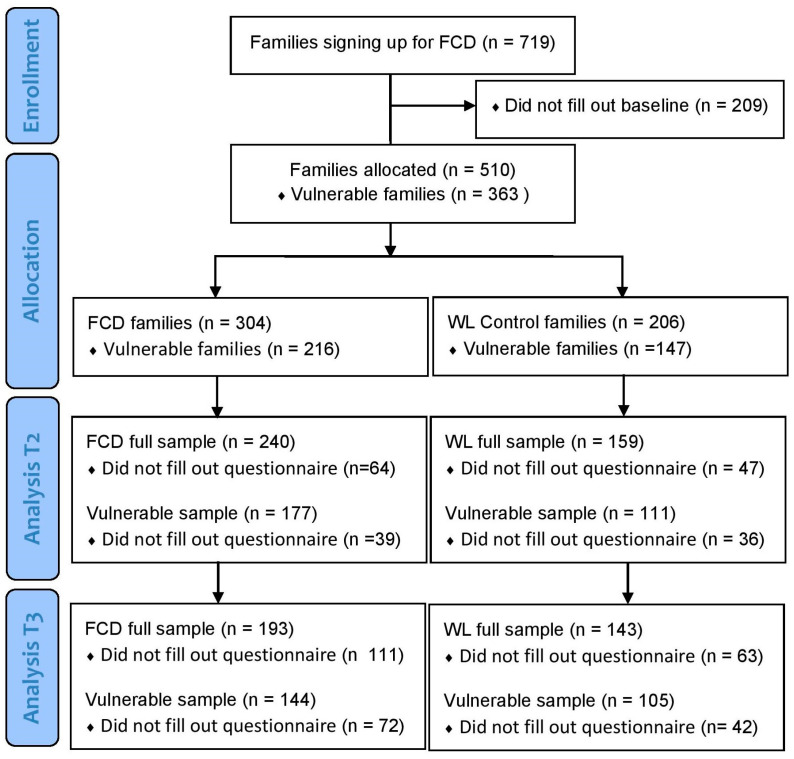
Trial flowchart.

A total of 719 families signed up for FCD and agreed to participate in the trial. A total of 209 families never completed the baseline questionnaire, leaving 510 families in the full sample. The study flow is presented in Figure 1.

### 3.1. Participants

The participants comprised 510 families (206 wait list and 304 FCD) representing 157 different FCDs. Based on baseline characteristics, 363 (71%) of the sample were identified as vulnerable families (206 wait list and 304 FCD). Most vulnerable families were characterized by low income, small social networks, low levels of support, lack of contact with other adults, physical or mental health problems, or being single parents.

The majority of volunteers were women with an average age of 51 years (range 30–79). Roughly half were either in education or employment, while one in four were retired, and 7% were not retired or engaged in education or employment. 

On average, families participated in 5.8 sessions, though there was significant variation in participation before and after the COVID-19 pandemic. Families who participated in FCD before the pandemic and were not affected by it attended an average of seven sessions. In contrast, families who participated during and after the pandemic attended an average of only 3.2 sessions.

Means, standard deviations, and *p*-values for comparisons for the baseline measures for the full sample (N = 510) and the vulnerable sample (N = 363) are shown in Table 2. Respondents are primarily women, and almost half are from single-parent households. Less than half of the respondents are in employment, and about one-fourth have high school as their highest level of education completed. About 25% of the families primarily speak a language different from Danish in their home.

We found only minor differences when comparing the mean values of the FCD and wait list groups at the baseline. Specifically, we observed that (1) FCD families more often had a male respondent, (2) FCD families reported fewer conflicts with their partners, and (3) FCD families felt more comfortable playing with their children. These differences were consistent across both the full sample and the vulnerable sample. Additionally, in the vulnerable sample, FCD families had more children compared to wait list families, and in the full sample, FCD families reported slightly greater access to practical help than wait list families. Out of forty-one imbalance checks, four were statistically significant, which is slightly more than expected by chance. However, these imbalances were related to single items rather than scales, and the magnitude of the differences was small.

### 3.2. Attrition from the Study at T2

The full and the vulnerable samples are reduced by 21% from the baseline to the assessment 6 months after (from 510 to 399 for the full sample and from 363 to 288 for the vulnerable sample). The results are shown in Supplementary Table S1. For both the full and vulnerable samples, dropout from the assessment after 6 months is significantly associated with reporting fewer conflicts between child and parent and missing information on education level or labor market status (meaning that they did not complete the baseline questionnaire). For the full sample, dropout is also associated with the father responding to the questionnaire. For the vulnerable sample, significantly more control families dropped out (there is also a trend for this in the full sample). Unsurprisingly, parents who did not complete the baseline questionnaire were more likely to not respond to the following assessment. With 44 tests completed, the number of imbalances is not worrying.

### 3.3. The Effects of FCD at Post-Intervention (T2) 

#### 3.3.1. Vulnerable Sample 

The primary analysis was conducted on the 363 families classified as vulnerable, as we expected that these families had a larger potential for change. The results of the effect analysis are displayed in Table 3.

We find a significant effect of FCD in three outcomes: FCD parents report that their children have a more challenging time forming friendships compared to control parents (*p* = 0.01, [−0.51;−0.09], *d* = 0.29), FCD parents experience less need for help to play with their children (*p* = 0.01, [−0.34;−0.05], *d* = 0.33), and FCD parents feel more confident when playing with their children (*p* = 0.04 [0.01; 0.40], *d* = 0.25) compared to control parents. 

#### 3.3.2. Full Sample

We repeated the analysis for the full sample to explore whether vulnerable families experience larger effects than the full sample (see Supplementary Table S2). For the full sample, the results correspond to the vulnerable sample. Since the results from the full sample mirror those of the vulnerable sample, we use the full sample for the remaining subgroup analyses to gain statistical power.

### 3.4. Robustness 

We conducted analyses based on imputed data to examine the robustness of the results (see Supplementary Table S3). Comparing the results of this analysis with the primary analysis, we observe consistent patterns, underscoring the reliability and validity of the results concerning attrition bias.

### 3.5. Progression over Time for FCD Families 

All families can choose to participate in FCD in a second round after finishing the first round. Consequently, results assessed at the 12-month follow up cannot be interpreted as causal effects. Therefore, we examine changes from T2 to T3 for FCD families to see how the group develops over time (see Supplementary Table S4).

For FCD families, we see the following changes from T2 to T3: FCD children participate significantly more in leisure activities (*p* = 0.01) at T3 than at T2. Similarly, there is a trend for FCD parents to participate more in leisure activities (*p* = 0.06) at T3 compared to T2. 

Some families participated in FCD in both periods. These families do not differ at T2 from families who only participated in one round of FCD. We, therefore, compare families who participate in one versus two rounds of FCD. At the 12-month follow up, we find that parents participating in two rounds of FCD have significantly fewer conflicts with their children than parents participating in one round (*p* = 0.02), access to less practical help (*p* = 0.05), and FCD children participating in two rounds of FCD more easily form new friendships than children participating in one round (*p* = 0.03) (see Supplementary Table S4).

### 3.6. Differential Effects

We conduct subgroup analyses to examine potential differences between subsets of the following participants: family composition (single parents or cohabiting parents); the age of the target child (<eight years old or ≥eight years old); the number of sessions attended (dose); and COVID-19 (see Table 4).

#### 3.6.1. Family Composition and Child Age

Almost 50% of the families participating in FCD evaluation are one-parent households. When comparing the results for one-parent households with cohabiting parents, we find that one-parent FCD families experience more frequent conflicts with their (non-cohabiting) partner (*p* = 0.08). On the contrary, the reverse is observed for cohabiting FCD families (though not significantly). When we compare results for younger children (2–7 years old) with older children (8 years or older), we find that conflicts between children and parents are reduced for the older FCD children compared to control families at T2 (*p* = 0.03). 

#### 3.6.2. Participation in FCD (Dose)

Families’ participation in FCD varied, leading to different exposure levels. Each family independently decided whether to participate in individual FCD sessions, and the number of sessions fluctuated due to COVID-19. Consequently, any association between FCD participation and outcomes cannot be interpreted as causal. Instead, it suggests a correlation between participation levels and outcomes, potentially capturing both treatment effects and selection bias. We examine the relationship between intervention dosage (wait list, 1–4 times participation, five times or more participation) and outcomes. We find that higher participation rates are significantly associated with lower self-worth (*p* = 0.03), lower parental stress (*p* = 0.03), and improved child health (*p* = 0.03). 

#### 3.6.3. COVID-19 

The COVID-19 pandemic arrived in Denmark during the study period and caused several changes to this study. From March 2020 to September 2021, Denmark experienced several lockdowns, assembly restrictions, and other restrictions that affected how FCD was carried out. During periods of lockdown, many FCDs closed down, and others changed to a shorter three-session online version with takeaway meals. In periods following lockdowns, FCD was continued with smaller groups, outside activities, and other accommodations to comply with restrictions. We have examined whether the interruption by COVID-19 gives rise to different effects of FCD by comparing the effects for families (FCD and control) who responded to the baseline survey before March 2020 (and who were not affected by COVID-19) with families who responded to the baseline questionnaire after March 2020 (and were affected by COVID-19). Results are presented in Table 4. 

We find that families participating in the original setup of FCD before COVID-19 initiated more play with their children (*p* = 0.04) compared to control families. In the main analysis, we found that families participating in FCD experience a reduced need for help playing with their children. The COVID-19 analysis reveals that this effect is mainly present among families participating in FCD during and after COVID-19 (*p* = 0.01). On the contrary, the main effect that FCD parents felt more comfortable playing with the child is mainly present among families participating in FCD before COVID-19 (*p* = 0.02). The third main effect where parents report that the child is having more difficulty forming friendships after participating in FCD is present in families participating both before and during COVID-19. 

### 3.7. Parent’s and Children’s Experience Participating in FCD

Three out of four participating parents reported having a positive experience with their family club. They felt at ease with their fellow parents, that their opinions were valued, and were confident in their ability to contribute to the community. These parents emphasized the importance of community and unity, engaging in activities, building networks, sharing meals, and enjoying various activities with their children, including outdoor adventures. Around half of the parents found inspiration for activities and cooking from their family club, and some reported making new acquaintances through it. The children were also satisfied with spending quality time with their parents and enjoyed being part of a family club. About one-third of parents reported that their children had made new friends through the family club. Three out of four families wished to continue participating in a family club. For families with limited social networks, involvement in a family club significantly enhanced their sense of belonging and recognition within the community. Conversely, for less vulnerable families, participating in a family club was primarily seen as a positive contribution to strengthening the cohesion of the local community.

### 3.8. Volunteer’s Experience Participating in FCD

Two-thirds of the volunteers underwent training, with almost all of them feeling adequately prepared to fulfill their responsibilities in practice. Moreover, a substantial majority (66%) are involved in additional volunteer activities beyond their commitment to FCD, demonstrating a strong dedication to community service.

Based on the qualitative data, we categorized the volunteers into four types: supermom, outdoor enthusiast, paid employee, student, and retiree. 

Volunteers expressed diverse motivations for their involvement in FCD, with many emphasizing the importance of having fun and making a positive impact on others’ lives. Social connections, skill development, and personal growth were also valued, while fewer prioritized career advancement through volunteering. Notably, several volunteers highlighted the valuable learning opportunities provided by their volunteer work, particularly in areas such as leadership, organization, and group dynamics. For some, participating in FCD also served as their first experience speaking in front of a group.

Beyond personal development, involvement in the family club fostered a sense of community and strengthened personal networks for some volunteers. Witnessing the growth of families and forming new acquaintances brought joy and fulfillment to their volunteer experience. This positive impact is reflected in the desire of more than half of the volunteers to continue their involvement with FCD, underscoring the meaningful connections and experiences cultivated through their participation.

### 3.9. Lessons Learned and Paths Forward

We identified several challenges that could provide valuable insights for future family clubs and similar initiatives. One such challenge was the inconsistent participation of some families, which led to frustration for both volunteers and peer families. Several volunteers found it challenging to retain families, highlighting the extensive effort required for planning and preparation, especially when families frequently canceled last minute or did not show up. This inconsistency made it difficult to ensure the smooth running of activities and affected the overall operation of the family club. It is crucial to set realistic expectations and make accommodations for families who may not be able to participate as consistently as others due to various reasons, especially considering the challenges faced by vulnerable families. Additionally, volunteers may require assistance and guidance when supporting particularly vulnerable families, recognizing the additional complexities and needs that may arise in these situations.

Furthermore, there is a necessity to provide support for volunteers to effectively balance the participation of both vulnerable and non-vulnerable families. This equilibrium is crucial to cultivating and maintaining openness and inclusivity within the family clubs, ensuring that all members feel valued and included regardless of their background or circumstances.

Lastly, there is a pressing need for increased focus on fathers or male participants within the family club. Some fathers expressed feeling somewhat on the periphery in this predominantly female environment, highlighting the importance of creating a welcoming and inclusive atmosphere for all participants. 

## 4. Discussion

This study is a prospective quasi-experimental trial assessing the effects of the volunteer intervention FCD on parents and their children compared to a wait list control group. We find no significant effect on the primary outcome or parent well-being for both the full sample and the vulnerable group. However, regarding secondary outcomes, FCD parents report feeling more confident when engaging in playtime with their children and requiring less assistance playing with them. Additionally, they report that their children have a more challenging time forming friendships compared to control parents. The effects are consistent across the two samples, are small-to-moderate (according to Cohen), are stable over time, and the results are robust to the estimation method.

Notably, vulnerable and non-vulnerable families experience improved playtime with their children by participating in FCD. This result is potentially significant since children learn through play [55]. Spending quality time engaged in play may also improve the parent–child relationship. The improvement in playtime between parents and children may be attributed to the volunteer-based nature of FCD as opposed to a standard service offered by, e.g., a municipality. A realist review of lay health worker interventions in the UK found that parents perceive laypersons as peers, which can foster positive engagement and establish trust, ultimately empowering parents to enhance their parenting skills over time [56]. However, the reasons behind FCD parents reporting that their children face greater challenges in forming friendships compared to control group parents remain unclear. One plausible explanation is that parents, as a result of their involvement in FCD, have become more attuned to any potential friendship-related issues their children might be experiencing. 

When we examine the changes over time for the families who participate in FCD, we find that children (and possibly parents) participate significantly more in leisure activities at the 12-month assessment than at the 6-month assessment. If participation in FCD is an alternative to other leisure activities, this outcome aligns with expectations, particularly as families gradually discontinue their involvement in FCD. However, it is essential to consider that families’ decisions to participate in FCD are selective and could be influenced by unobserved family characteristics. Therefore, any observed differences between participating families should be interpreted as associations rather than a direct causal effect of participating in FCD.

The mixed results of this study align reasonably with previous research on volunteer family support programs in developed countries. While certain studies have identified positive effects [25,27,30], others have reported mixed results or no discernible effects [28,29,37,38].

### 4.1. Differential Effects of FCD 

We have examined whether the effects of FCD vary for different groups. We only find a few unsystematic effects for family composition and child age, which may be interpreted as spurious effects considering the number of tests. When examining correlations between high participation in FCD and outcomes, we find that families with low self-worth are likelier to participate in more sessions. However, we also find that high participation significantly correlates with low parental stress and improved child health. Together, the results indicate that we do not find systematic differences in the effects of FCD. 

### 4.2. FCD and COVID-19

The COVID-19 pandemic posed substantial challenges to all trials, affecting various aspects such as recruitment, retention, and intervention. Lockdowns and assembly restrictions disrupted the enrollment of new families to this trial and challenged data collection at the post-intervention and follow-up stages. Throughout 2020 and 2021, the pandemic imposed significant restrictions, necessitating innovative approaches to conduct family clubs in this new reality. As a result, FCD evolved from its initial format of twelve face-to-face sessions where families dined and engaged in activities together to smaller outdoor groups or a condensed three-session online version with takeaway meals. Apart from its impact on the execution of FCD, it is reasonable to assume that COVID-19 also had significant individual effects on the participating families due to their different work situations, financial stability, potential need for homeschooling, and health statuses. Many volunteers and families also experienced heightened health concerns, leading some to choose isolation. These circumstances likely influenced the well-being of the families involved, but we could not account for these factors in our analyses. 

When comparing the results of families who participated in FCD before COVID-19 with those who participated during the pandemic, we find that two of the three main effects are influenced by COVID-19. The increase in parenting confidence while playing with their children is primarily observed in families who participated in the original FCD setup before the pandemic. Conversely, the reduced need for help with playing is mainly observed in families who participated during the COVID-19 period. This suggests that the two distinct FCD setups (the large in-person twelve-session group and the virtual three-session group) may lead to different outcomes, maybe because they attract different types of families. The third main effect, however, where parents report that their children have more difficulty forming friendships after participating in FCD, is present in families participating both before and during COVID-19.

#### 4.2.1. Navigating Challenges

Navigating the terrain of volunteer interventions presents significant challenges, as the inherent nature of voluntary engagement often clashes with the requirements for conducting robust assessments. Volunteers’ behaviors and actions are driven by their individual perspectives and sometimes diverge from a standardized intervention protocol. While the intervention concept in our project was standardized, and volunteers underwent a one-day training, the success of the intervention heavily relied on the engagement and personal qualities of the volunteers. Acknowledging the impracticality and undesirability of enforcing rigid adherence to a manualized approach, this study was approached with flexibility, respecting local needs and variations. Consequently, while practical implementation was standardized as much as possible, the actual frequency and specific activities of each family club varied considerably. Additionally, family clubs were hosted in diverse settings with varying characteristics, including levels of ethnic diversity, prevalence of public housing, and disparities in family income, potentially introducing variations in both the delivery and reception of FCD activities. The volunteers in FCD did not necessarily have a professional background and did not receive thorough training, as is the case in some of the other studies of volunteer interventions demonstrating positive effects of the intervention [25,57]. 

#### 4.2.2. Insights for Future Family Support Interventions

Across observations, interviews, and questionnaire comments, both families and volunteers expressed high satisfaction with their participation in FCD. Similarly, previous studies of volunteer family support programs in developed countries have found that parents express satisfaction with the support when surveyed [25,27]. However, it appears that none of these studies collected data on satisfaction through interviews or observations. This aligns with a review indicating that only 37% of trials included in a Cochrane review on the use of lay health workers incorporated qualitative research [58]. The review concludes that there is a dearth of qualitative research to illuminate results from effectiveness trials. When qualitative data were included in a trial, a recurring theme was the participants’ experiences. Qualitative studies conducted alongside trials of complex interventions are crucial and can provide insights into processes, contextual factors, or intervention characteristics that may influence quantitative results. Therefore, it is essential to include qualitative data in future trials. 

The observations and interviews conducted in this study offered insights into issues that could be pertinent for future family clubs and similar interventions. Firstly, it is crucial to adjust expectations and tailor the intervention to accommodate families who, for various reasons, may not be able to participate as consistently as others. These families likely need the intervention the most. Secondly, volunteers may require assistance and guidance in supporting particularly vulnerable families, as these parents may face severe mental health issues and challenges in parenting their children. This guidance could also extend to supporting families with different cultural or religious values. Thirdly, there may be a need to support volunteers in balancing the participation of vulnerable and non-vulnerable families in the family club, as inclusion may be compromised if there are too few or too many non-vulnerable families. Lastly, there is a need for increased attention towards fathers or male participants in family clubs.

### 4.3. Limitations

One study limitation is the lack of individual randomization. Participants were primarily assigned to FCD on a first-come, first-served basis, potentially introducing selection bias if early responders differ from later participants regarding enthusiasm or other characteristics. However, our recruitment strategy employed multiple channels targeting potential families in diverse ways. Combining personal recruitment by municipal social workers or local social housing employees with an online sign-up process possibly mitigated the potential bias with sign-up timing. In addition, first responders could be assigned to the control group for other reasons, such as no club in their local area or scheduling conflicts preventing them from participating on the offered day, introducing an element of randomness in the allocation. Therefore, we expect the potential selection issues introduced by the first-come, first-served principle to be minimized.

Another limitation is the relatively high dropout rate observed through this study. Although we initially recruited twice the expected number of participants (510 compared to 250) at the baseline, this number decreased to 399 at the post-intervention stage, resulting in an attrition rate of 22%. However, it is worth noting that our study’s statistical power to detect differences between the two groups remains intact because we initially recruited a larger sample at the baseline.

Finally, we had limited control over how the intervention was implemented, which is inherently challenging when the intervention relies on volunteers. The changes necessitated by the COVID-19 pandemic introduced further variation in how the intervention was carried out. This variability may pose challenges when attempting to generalize the findings from FCD to other settings. Nevertheless, it is important to recognize that our study was conducted as a pragmatic trial within a naturalistic setting. This approach can be considered a strength as it reflects the real-world context in which FCD is typically applied.

## 5. Conclusions

This study evaluated the FCD volunteer intervention’s effects on parents and their children compared to a wait list control group. While there was no significant effect on the primary outcome of parent well-being, FCD parents reported increased confidence in playtime and needing less assistance compared to control parents. They also noticed their children had more difficulty forming friendships compared to the control group. These small-to-moderate effects, consistent over time, suggest that both vulnerable and non-vulnerable families benefit from improved parent–child playtime through FCD. The COVID-19 pandemic significantly impacted the trial, altering the FCD format and influencing family experiences. Families in the original 12-session in-person setup before the pandemic showed increased confidence during playtime compared to the control group. Those in the pandemic’s virtual three-session format reported needing less help with play compared to the control group. This suggests that different FCD setups might yield varied outcomes. 

## Figures and Tables

**Table 1:** Timing of measures.

Parent Measures		T1	T2	T3
Background	Age, gender, language, education	√	√	√
Family	Partner, children	√	√	√
Mental health	Warwick–Edinburgh Mental Well-being Scale	√	√	√
Self-efficacy	From the general self-efficacy scale	√	√	√
Parental stress	Parental Stress Scale	√	√	√
Family life	Leisure activities	√	√	√
Family routines	Mealtime, duties, bedtime, homework	√	√	√
Network	Loneliness, practical help, confidants	√	√	√
Parenting competences	From Parent Behavior Inventory	√	√	√
Play	Play with children	√	√	√
Screen time	Parent	√	√	√
Family budget	Worries, budget	√	√	√
Satisfaction	Participation, network		√	
Child measures		T1	T2	T3
Well-being child (<6)	Well-being	√	√	√
Well-being child (≥6)	KIDSCREEN-10	√	√	√
Network	Friends	√	√	√
Screen time	Mobile phone, computer	√	√	√
Learning activities	Reading, talking	√	√	√

**Table 2:** Demographic characteristics for the full and vulnerable samples at the baseline (T1) for families who participated in FCD and wait list controls.

	Full Sample						Vulnerable Sample					
	WL N = 206		FCD N = 304		Chi-2/t	*p*	WL N = 147		FCD N = 216		Chi-2/t	*p*
	Mean/%	SD	Mean/%	SD			Mean/%	SD	Mean/%	SD		
Vulnerable	71.36%		71.05%		0.01	0.94						
Female	95.63%		90.46%		4.76	0.03	98.64%		93.98%		4.79	**0.03**
Age	38.07	(6.80)	37.00	(7.45)	1.50	0.13	38.07	(7.01)	37.11	(7.39)	1.15	0.25
Shares household with partner	52.45%		55.48%		0.45	0.50	46.58%		50.23%		0.47	0.50
Number of children	1.88	(0.85)	2.02	(0.93)	−1.79	0.07	1.81	(0.86)	2.05	(0.92)	−2.47	**0.01**
Danish spoken most at home	76.44%		71.21%		1.46	0.23	74.40%		72.31%		0.17	0.68
*Occupation status*												
Employed	46.12%		40.79%		6.44	0.17	37.41%		31.94 %		8.36	0.08
In education	7.77%		9.87%				4.76%		7.4%			
On medical leave/unemployed with benefits	11.65%		8.88%				16.33%		12.50%			
Retired, social security, stay at home, leave, or other	18.93%		27.30%				26.53%		38.43%			
Missing information	15.53%		13.16%				14.97%		9.72%			
*Education level*												
High school or lower	16.50%		25.00%		6.62	0.09	19.05%		29.63%		7.28	0.06
Short or vocational education	27.18%		28.62%				27.89%		29.63%			
Higher education	40.78%		33.22%				38.10%		31.02%			
Missing information	15.53%		13.16%				14.97%		9.72%			

Bold items are significant at *p* < 0.05; WL: wait list control; FCD: Family Club Denmark; t: *t*-test; SD: standard deviation.

**Table 3:** Comparison of parent and child outcomes for vulnerable FCD and wait list families at the baseline (T1) and after 6 months (T2) with regression coefficients, 95% confidence intervals, *p*-values, and effect sizes for multiple linear regressions with controls.

	T1				T2				B	CI	*p*	d
	WL N = 206		FCD N = 304		WL N = 159		FCD N = 240					
	Mean	SD	Mean	SD	Mean	SD	Mean	SD				
Well-being	21.21	(3.02)	21.92	(3.89)	21.90	(3.28)	22.50	(4.12)	0.21	[−0.50, 0.92]	0.56	0.05
Self-efficacy	9.19	(1.96)	9.20	(1.90)	9.48	(1.80)	9.21	(1.98)	−0.19	[−0.52, 0.14]	0.27	−0.10
Self-worth	10.00	(2.42)	10.02	(2.77)	10.50	(2.49)	10.52	(2.69)	0.18	[−0.38, 0.75]	0.52	0.07
Lack of parental satisfaction	10.78	(3.23)	10.46	(3.87)	11.27	(3.97)	10.80	(4.27)	−0.11	[−1.02, 0.79]	0.81	−0.03
Parental stress	17.28	(4.78)	17.29	(5.02)	17.42	(4.82)	17.39	(4.85)	−0.10	[−1.08, 0.87]	0.83	−0.02
Family routines	42.21	(4.67)	42.37	(4.50)	43.05	(3.76)	42.50	(4.39)	−0.46	[−1.33, 0.41]	0.30	−0.11
Supportive PB	26.47	(2.78)	26.91	(2.72)	27.01	(2.64)	26.69	(3.07)	−0.41	[−0.98, 0.15]	0.15	−0.14
Hostile PB	13.28	(3.61)	13.23	(3.30)	12.86	(3.66)	13.03	(3.10)	0.05	[−0.68, 0.78]	0.88	0.02
Network: practical help	2.68	(1.18)	2.99	(1.35)	2.92	(1.18)	2.96	(1.28)	−0.16	[−0.42, 0.09]	0.21	−0.13
Network: confidants	3.62	(1.19)	3.72	(1.24)	3.79	(1.15)	3.76	(1.23)	−0.03	[−0.29, 0.22]	0.79	−0.03
Network: loneliness	3.31	(1.06)	3.22	(0.96)	3.29	(0.87)	3.11	(1.05)	−0.12	[−0.31, 0.07]	0.22	−0.12
Parents: conflicts with child/children	3.73	(1.82)	3.38	(2.08)	3.95	(1.76)	3.46	(1.88)	−0.30	[−0.66, 0.06]	0.10	−0.16
Parents: conflicts with partner *	2.57	(1.79)	1.87	(1.69)	2.38	(1.85)	1.89	(1.79)	−0.11	[−0.57, 0.35]	0.63	−0.06
Parent: participation in leisure activities	0.25	(0.43)	0.31	(0.46)	0.27	(0.44)	0.29	(0.46)	−0.02	[−0.13, 0.09]	0.73	−0.05
Feel comfortable playing with child/children *	4.00	(0.82)	4.37	(0.72)	3.93	(0.94)	4.25	(0.71)	0.21	[0.01, 0.40]	**0.04**	0.25
Need help to play with child/children	1.69	(0.62)	1.53	(0.68)	1.66	(0.63)	1.43	(0.60)	−0.20	[−0.34, −0.05]	**0.01**	−0.33
Initiates playtime with child/children	0.11	(0.32)	0.09	(0.29)	0.12	(0.33)	0.11	(0.31)	−0.03	[−0.11, 0.05]	0.50	−0.09
Parents: screen time	5.27	(2.09)	4.96	(2.26)	5.16	(2.15)	4.85	(1.98)	−0.07	[−0.48, 0.33]	0.73	−0.03
Learning activities	14.50	(2.86)	14.52	(2.83)	14.79	(3.41)	14.45	(2.97)	−0.22	[−0.82, 0.39]	0.48	−0.07
Frequency of family dinners	3.56	(0.67)	3.68	(0.64)	3.60	(0.66)	3.64	(0.60)	−0.00	[−0.14, 0.14]	0.96	−0.01
Parents: easiness of forming friendships	2.55	(1.00)	2.80	(1.10)	2.79	(1.12)	2.95	(1.16)	0.00	[−0.23, 0.23]	0.99	0.00
Well-being (child age > 8 years)	43.93	(10.61)	42.83	(9.01)	43.07	(11.81)	42.82	(9.25)	1.19	[−3.23, 5.62]	0.59	0.12
Well-being (child age < 8 years)	33.60	(3.26)	34.15	(4.15)	33.46	(3.94)	33.22	(3.92)	−0.13	[−1.49, 1.23]	0.85	−0.03
Child: conflicts with parents	3.09	(0.93)	2.92	(0.95)	3.16	(0.85)	2.85	(0.96)	−0.15	[−0.37, 0.06]	0.16	−0.17
Child: conflicts with peers	2.67	(0.90)	2.57	(0.84)	2.58	(0.76)	2.53	(0.77)	0.05	[−0.12, 0.22]	0.58	0.07
Child: participation in leisure activities	0.61	(0.49)	0.51	(0.50)	0.54	(0.50)	0.48	(0.50)	−0.04	[−0.17, 0.09]	0.57	−0.07
Health of child	3.64	(0.85)	3.95	(0.71)	3.60	(0.97)	3.76	(0.83)	−0.12	[−0.61, 0.37]	0.63	−0.13
Child has sleep problems	2.21	(0.99)	2.04	(0.95)	2.08	(1.00)	2.10	(0.95)	0.20	[−0.00, 0.41]	0.06	0.21
Child has one or more close friends	0.75	(0.44)	0.87	(0.34)	0.80	(0.40)	0.92	(0.27)	0.09	[−0.01, 0.18]	0.06	0.26
Child: screen time	3.55	(1.12)	3.72	(1.09)	3.81	(1.13)	3.85	(1.14)	0.08	[−0.13, 0.30]	0.44	0.07
Child: easiness of forming friendships	3.75	(1.05)	3.77	(0.97)	3.92	(1.08)	3.67	(1.00)	−0.30	[−0.51, −0.09]	**0.01**	−0.29

Bold items are significant at *p* < 0.05; T1: Time 1 (baseline); T2: Time 2 (after 6 months); b: regression estimate, CI: 95% confidence interval; WL: wait list control; FCD: Family Club Denmark; SD: standard deviation. * imbalance at the baseline *p* < 0.05.

**Table 4:** Regression results of interaction analyses for family composition, child age, dosage, and COVID-19.

	One-Parent Household vs. Cohabiting		<8 Years vs. 8+ Years		<4 vs. 4+ Sessions		Before COVID-19 vs. during and after COVID-19	
Test for Difference	b	*p*	b	*p*	b	*p*	b	*p*
Well-being	0.61	0.37	0.01	0.99	0.34	0.46	−0.20	0.78
Self-efficacy	0.26	0.38	−0.06	0.87	0.22	0.31	0.23	0.44
Self-worth	0.18	0.71	0.18	0.75	−0.74	**0.03**	0.25	0.62
Lack of parental satisfaction	0.42	0.11	0.08	0.78	0.24	0.14	−0.33	0.19
Parental stress	0.48	0.19	0.51	0.22	−0.52	**0.03**	0.40	0.26
Family routines	−0.21	0.80	−1.16	0.19	0.50	0.28	0.70	0.38
Supportive PB	0.23	0.67	0.08	0.89	−0.01	0.97	−0.35	0.50
Hostile PB	−0.63	0.33	1.03	0.13	−0.50	0.19	−0.55	0.40
Network: practical help	−0.36	0.13	−0.22	0.41	0.05	0.74	−0.32	0.16
Network: confidants	0.25	0.23	0.16	0.49	0.13	0.32	−0.32	0.14
Network: loneliness	0.08	0.67	−0.09	0.64	−0.12	0.35	−0.03	0.89
Parents: conflicts with child/children	0.02	0.94	0.29	0.35	0.27	0.19	−0.37	0.24
Parents: conflicts with partner	1.51	**0.01**	−0.14	0.79	−0.32	0.21	−0.30	0.42
Parent: participation in leisure activities	−0.03	0.75	0.02	0.86	−0.02	0.73	−0.00	0.97
Feel comfortable playing with child/children	−0.05	0.77	−0.03	0.87	−0.08	0.43	−0.17	0.33
Need help to play with child/children	−0.04	0.75	−0.01	0.90	0.03	0.69	−0.12	0.34
Initiates playtime with child/children	−0.05	0.48	0.04	0.58	0.04	0.34	−0.11	**0.04**
Parents: screen time	−0.61	0.08	0.59	0.12	0.32	0.14	−0.20	0.58
Learning activities	0.01	0.98			0.22	0.53	−0.77	0.18
Frequency of family dinners	0.09	0.48	−0.00	0.98	−0.03	0.75	0.13	0.29
Parents: easiness of forming friendships	0.27	0.15	0.10	0.65	−0.20	0.11	−0.18	0.38
Well-being (child age > 8 years)	0.52	0.92			−0.40	0.88	4.91	0.66
Well-being (child age < 8 years)	−2.75	0.06			−0.72	0.36	0.00	.
Child: conflicts with parents	−0.04	0.83	0.38	**0.03**	0.08	0.49	0.09	0.63
Child: conflicts with peers	−0.10	0.51	0.15	0.33	0.08	0.46	−0.05	0.72
Child: participation in leisure activities	0.15	0.17	0.02	0.85	−0.03	0.59	−0.07	0.51
Health of child	0.72	0.16			0.59	**0.03**	2.07	**0.01**
Child has sleep problems	0.14	0.45	−0.07	0.69	−0.20	0.08	0.11	0.55
Child has one or more close friends	0.12	0.11	−0.04	0.61	−0.02	0.69	0.06	0.45
Child: screen time	0.25	0.18	0.29	0.16	−0.00	0.97	−0.16	0.42
Child: easiness of forming friendships	−0.18	0.36	−0.28	0.14	−0.12	0.28	0.10	0.58

Bold items are significant at *p* < 0.05; b: regression estimate.

## Data Availability

The datasets generated and analyzed during the current study are not publicly available to protect participant privacy but are available from the corresponding author upon reasonable request.

## Supplementary Materials

The following supporting information can be downloaded at https://www.mdpi.com/article/10.3390/healthcare12111115/s1. Table S1: Attrition analyses for the full and vulnerable sample at 6 months (T2). Table S2: Comparison of parent and child outcomes for the full sample of FCD and wait-list families at baseline (T1) and after 6 months (T2) with regression coefficients, 95 % confidence intervals, *p*-values, and effect sizes for multiple linear regressions with controls. Table S3: Robustness analyses for the full sample and vulnerable sample comparing parent and child outcomes for FCD and wait-list control families at 6 months (T2) with regression coefficients, 95 % confidence intervals, and *p*-values for multiple linear regressions on imputed data with controls. Table S4: Means for FCD families with one or two FCD rounds at 6 months (T2) and 12 months (T3). Comparison of all FCD families from T2 to T3 and comparison of families with one versus two rounds of FCD.
