# Supplementary material for: Family Club Denmark: A Quasi-Randomized Study of a Volunteer-Based Intervention to Support Vulnerable Families
Source: Healthcare (Basel). 2024 May 29;12(11):1115. doi: 10.3390/healthcare12111115 (PMC11171909; doi:10.3390/healthcare12111115)
Supplement: Supplementary file 1 [file healthcare-12-01115-s001.zip › healthcare-3007868-supplementary.pdf]

healthcare

MDPI

Supplementary Table S1. Attrition analyses for the full and vulnerable sample at 6 months (T2).

	Full sample		Vulnerable sample	
	Dropout at 6 months		Dropout at 6 months	
	Coefficient	P-value	Coefficient	P-value
Treatment group	-0.071	0.083	-0.10	**0.05**
Female	-0.165	**0.047**	-0.22	0.10
Vulnerable family	-0.043	0.330		
Age of parent	-0.002	0.361	-0.00	0.64
Age of child	0.002	0.732	0.01	0.15
Lives alone	-0.034	0.384	0.02	0.66
Number of children	0.009	0.683	0.03	0.35
Employed	0.000		0.00	
In education	0.002	0.981	-0.05	0.59
Medical leave/Unemployed	-0.041	0.511	-0.07	0.30
Early retirer/social security recipient or other	-0.065	0.146	-0.10	0.07
Missing information	0.360	**0.000**	0.28	**0.00**
High school or lower	0.000		0.00	
Short or vocational education	-0.036	0.477	-0.05	0.43
Higher education	0.020	0.697	0.01	0.90
Missing information	0.379	**0.000**	0.32	**0.00**
Primary language in home not Danish	0.022	0.626	0.02	0.67
Self-perceived financial status	-0.001	0.964	-0.00	0.84
Financial worries	0.003	0.849	0.01	0.45
Well-being	0.005	0.310	0.00	0.44
Self-efficacy	0.007	0.479	0.01	0.59
Self-worth	0.013	0.058	0.01	0.23
Lack of parental satisfaction	-0.005	0.784	-0.01	0.64
Parental stress	0.015	0.153	0.02	0.13
Family routines	0.000	0.991	0.00	0.98
Supportive PB	0.004	0.574	0.01	0.40
Hostile PB	-0.004	0.538	-0.00	0.71
Network: Practical help	0.001	0.932	-0.00	0.86
Network: confidants	0.018	0.228	0.03	0.07
Network: loneliness	-0.013	0.480	-0.02	0.48
Parents: Conflicts with child/children	-0.007	0.461	-0.01	0.55
Parents: Conflicts with partner	0.020	0.197	0.01	0.42
Parent: Participation in leisure activities	0.066	0.117	0.07	0.17
Feel comfortable in play with child/children	0.001	0.962	-0.00	0.89
Need help to playtime with child/children	-0.023	0.428	-0.03	0.31
Initiates playtime with child/children	0.061	0.369	-0.02	0.83
Parents: Screen time	-0.007	0.416	-0.01	0.34
Learning activites	-0.004	0.567	-0.01	0.54
Frequency of family dinners	-0.011	0.720	-0.01	0.80
Parents: Easiness of forming friendships	0.031	0.063	0.03	0.15
Well-being (child age > 8 years)	-0.001	0.839	0.00	0.73
Well-being (child age < 8 years)	0.008	0.218	0.01	0.38
Child: Conflicts with parents	-0.043	**0.036**	-0.05	**0.04**
Child: Conflicts with peers	0.021	0.394	0.03	0.26
Child: Participation in leisure activities	0.061	0.113	0.08	0.08
Health of child	-0.024	0.591	-0.04	0.48

Child has sleep problems	-0.017	0.415	-0.04	0.12
Child has one or more close friends	0.020	0.683	0.00	0.95
Child: Screen time	-0.014	0.462	0.00	0.82
Child: Easiness of forming friendships	0.005	0.815	-0.00	0.92
Covid-19 lock-down	-0.004	0.921	-0.03	0.56

Supplementary Table S2. Comparison of parent and child outcomes for the full sample of FCD and wait-list families at baseline (T1) and after 6 months (T2) with regression coefficients, 95 % confidence intervals, p-values, and effect sizes for multiple linear regressions with controls.

	T1				T2				b	CI	P	d
	WL N =		FCD N =		WL N =		FCD N =					
	Mean	SD	Mean	SD	Mean	SD	Mean	SD				
Well-being	22.16	(3.48)	22.69	(4.00)	22.58	(3.51)	23.10	(4.16)	-0.09	[-0.71, 0.54]	0.79	-0.02
Self-efficacy	9.50	(1.91)	9.51	(1.93)	9.64	(1.72)	9.55	(2.00)	-0.20	[-0.49, 0.08]	0.16	-0.11
Self-worth	10.58	(2.56)	10.50	(2.80)	10.87	(2.50)	10.68	(2.73)	-0.22	[-0.68, 0.24]	0.35	-0.08
Lack of parental satisfaction	0.56	(0.98)	0.63	(1.14)	0.74	(1.35)	0.62	(1.28)	-0.14	[-0.38, 0.10]	0.26	-0.11
Parental stress	2.98	(1.79)	3.10	(1.98)	2.99	(1.84)	3.18	(2.01)	0.02	[-0.32, 0.37]	0.89	0.01
Family routines	42.76	(4.36)	42.57	(4.42)	43.13	(3.68)	42.73	(4.27)	-0.15	[-0.88, 0.58]	0.68	-0.04
Supportive PB	26.75	(2.62)	26.99	(2.65)	26.91	(2.62)	26.77	(3.01)	-0.17	[-0.68, 0.35]	0.52	-0.06
Hostile PB	12.96	(3.66)	13.03	(3.24)	12.67	(3.65)	12.99	(3.28)	-0.14	[-0.77, 0.49]	0.66	-0.04
Network: Practical help*	2.83	(1.24)	3.13	(1.30)	2.99	(1.22)	3.14	(1.31)	-0.14	[-0.36, 0.07]	0.19	-0.11
Network: confidants	3.81	(1.21)	3.89	(1.24)	3.94	(1.15)	3.90	(1.22)	-0.10	[-0.29, 0.10]	0.35	-0.08
Network: loneliness	3.12	(1.10)	3.04	(1.00)	3.09	(0.94)	2.89	(1.09)	-0.08	[-0.25, 0.09]	0.33	-0.08
Parents: Conflicts with child	3.49	(1.85)	3.33	(2.10)	3.77	(1.78)	3.53	(1.82)	-0.02	[-0.31, 0.27]	0.87	-0.01
Parents: Conflicts with partner*	2.26	(1.76)	1.79	(1.65)	2.24	(1.76)	1.81	(1.81)	-0.20	[-0.57, 0.17]	0.29	-0.11
Parent: Participation in leisure activities	0.29	(0.46)	0.36	(0.48)	0.28	(0.45)	0.30	(0.46)	-0.03	[-0.13, 0.06]	0.49	-0.07
Feel comfortable in play with child*	4.13	(0.80)	4.34	(0.72)	3.97	(0.87)	4.25	(0.73)	0.20	[0.03, 0.36]	**0.02**	0.25
Need help to playtime with child	1.59	(0.62)	1.50	(0.64)	1.56	(0.62)	1.41	(0.58)	-0.12	[-0.24, -0.00]	**0.05**	-0.21
Initiates playtime with child/children	0.12	(0.33)	0.09	(0.28)	0.12	(0.32)	0.10	(0.30)	-0.00	[-0.06, 0.06]	0.89	-0.01
Parents: Screen time	5.18	(2.01)	4.98	(2.20)	5.01	(2.06)	4.89	(1.98)	0.06	[-0.29, 0.40]	0.75	0.03
Learning activites	14.68	(2.72)	14.65	(2.94)	15.00	(3.15)	14.50	(3.06)	-0.26	[-0.80, 0.28]	0.34	-0.08
Frequency of family dinners	3.56	(0.70)	3.64	(0.63)	3.64	(0.61)	3.69	(0.56)	-0.02	[-0.13, 0.10]	0.74	-0.03
Parents: Easiness of forming friendships	2.78	(1.16)	2.97	(1.12)	3.01	(1.19)	3.09	(1.15)	-0.07	[-0.25, 0.11]	0.44	-0.06
Well-being (child age > 8 years)	44.50	(10.05)	44.24	(9.08)	42.36	(10.62)	44.34	(9.55)	0.89	[-3.64, 5.41]	0.70	0.09

Well-being (child age < 8 years)	34.45	(3.51)	34.31	(3.93)	34.28	(3.99)	33.60	(3.59)	-0.57	[-1.80, 0.66]	0.36	-0.15
Child: Conflicts with parents	2.95	(0.94)	2.90	(0.95)	3.07	(0.84)	2.88	(0.95)	-0.09	[-0.27, 0.08]	0.30	-0.10
Child: Conflicts with peers	2.53	(0.82)	2.51	(0.83)	2.49	(0.75)	2.52	(0.77)	0.12	[-0.03, 0.26]	0.12	0.16
Child: Participation in leisure activities	0.61	(0.49)	0.52	(0.50)	0.54	(0.50)	0.48	(0.50)	-0.07	[-0.17, 0.03]	0.17	-0.14
Health of child	3.72	(0.90)	3.98	(0.69)	3.54	(1.00)	3.90	(0.82)	0.04	[-0.38, 0.45]	0.86	0.04
Child has sleep problems	2.02	(0.92)	2.04	(0.94)	1.97	(0.93)	2.05	(0.93)	0.12	[-0.06, 0.30]	0.19	0.13
Child has one or more close friends	0.78	(0.41)	0.85	(0.36)	0.82	(0.39)	0.89	(0.31)	0.03	[-0.04, 0.11]	0.38	0.10
Child: Screen time	3.49	(1.07)	3.61	(1.11)	3.65	(1.15)	3.72	(1.14)	0.16	[-0.02, 0.34]	0.08	0.14
Child: Easiness of forming friendships	3.85	(1.01)	3.81	(0.96)	3.94	(1.04)	3.68	(0.96)	-0.27	[-0.45, -0.09]	**0.00**	-0.28

Bold items are significant at p < 0.05; T1: Time 1 (baseline); T2: Time 2 (after 6 months); b: regression estimate, CI: 95 % confidence interval; WL: wait-list control; FCD: Family Club Denmark; SD: standard deviation. * inbalance at baseline p < 0.05

Supplementary Table S3. Robustness analyses for the full sample and vulnerable sample comparing parent and child outcomes for FCD and wait-list control families at 6 months (T2) with regression coefficients, 95 % confidence intervals, and p-values for multiple linear regressions on imputed data with controls.

	Full sample			Vulnerable sample		
	b	CI	P	b	CI	P
Well-being	0.15	[-0.42, 0.73]	0.60	0.28	[-0.41, 0.98]	0.42
Self-efficacy	-0.15	[-0.44, 0.14]	0.32	-0.21	[-0.56, 0.15]	0.25
Self-worth	-0.17	[-0.60, 0.27]	0.45	0.07	[-0.47, 0.62]	0.79
Lack of parental satisfaction	-0.35	[-1.03, 0.33]	0.31	-0.51	[-1.34, 0.33]	0.24
Parental stress	-0.05	[-0.85, 0.76]	0.91	0.19	[-0.79, 1.17]	0.71
Family routines	-0.03	[-0.77, 0.72]	0.94	-0.29	[-1.22, 0.64]	0.54
Supportive PB	-0.09	[-0.59, 0.41]	0.73	-0.42	[-1.01, 0.17]	0.16
Hostile PB	0.17	[-0.48, 0.83]	0.60	0.22	[-0.61, 1.05]	0.61
Network: Practical help	0.02	[-0.20, 0.24]	0.84	-0.01	[-0.27, 0.26]	0.97
Network: confidants	-0.07	[-0.27, 0.14]	0.54	-0.06	[-0.31, 0.20]	0.66
Network: loneliness	-0.17	[-0.36, 0.02]	0.08	-0.19	[-0.43, 0.05]	0.12
Parents: Conflicts with child	-0.10	[-0.40, 0.20]	0.51	-0.23	[-0.58, 0.12]	0.20
Parents: Conflicts with partner	-0.6	[-0.69, 0.16]	0.22	-0.22	[-0.78, 0.35]	0.44
Parent: Participation in leisure activities	-0.02	[-0.12, 0.07]	0.67	-0.01	[-0.13, 0.10]	0.80
Feel comfortable in play with child	0.24	[0.07, 0.41]	**0.01**	0.21	[-0.01, 0.42]	0.06

Need help to playtime with child	-0.13	[-0.25, -0.01]	**0.04**	-0.20	[-0.34, -0.05]	**0.01**
Initiates playtime with child	-0.02	[-0.08, 0.05]	0.61	-0.03	[-0.12, 0.06]	0.49
Parents: Screen time	0.10	[-0.26, 0.46]	0.58	-0.04	[-0.44, 0.35]	0.84
Learning activites	-0.35	[-0.89, 0.18]	0.20	-0.25	[-0.88, 0.39]	0.44
Frequency of family dinners	0.02	[-0.09, 0.14]	0.70	0.02	[-0.12, 0.16]	0.76
Parents: Easiness of forming friendships	-0.02	[-0.21, 0.18]	0.87	-0.04	[-0.28, 0.20]	0.73
Well-being (child age > 8 years)	0.79	[-4.59, 6.17]	0.77	-0.48	[-6.40, 5.45]	0.87
Well-being (child age < 8 years)	-0.73	[-2.03, 0.58]	0.27	-0.72	[-2.46, 1.02]	0.41
Child: Conflicts with parents	-0.13	[-0.28, 0.04]	0.13	-0.15	[-0.35, 0.06]	0.15
Child: Conflicts with peers	0.08	[-0.07, 0.24]	0.30	0.03	[-0.17, 0.23]	0.77
Child: Participation in leisure activities	-0.04	[-0.14, 0.06]	0.43	-0.03	[-0.16, 0.09]	0.61
Health of child	0.16	[-0.41, 0.73]	0.56	0.08	[-0.59, 0.74]	0.82
Child has sleep problems	0.10	[-0.08, 0.27]	0.28	0.16	[-0.05, 0.38]	0.14
Child has one or more close friends	0.06	[-0.02, 0.14]	0.17	0.10	[0.01, 0.19]	**0.04**
Child: Screen time	0.11	[-0.08, 0.30]	0.26	0.07	[-0.17, 0.31]	0.59
Child: Easiness of forming friendships	-0.25	[-0.44, -0.06]	**0.01**	-0.27	[-0.49, -0.05]	**0.02**

Bold items are significant at $p < 0.05$; b: regression estimate, CI: 95 % confidence interval

Supplementary Table S4. Means for FCD families with one or two FCD rounds at 6 months (T2) and 12 months (T3). Comparison of all FCD families from T2 to T3 and comparison of families with one versus two rounds of FCD.

		**6 month T2**			**12 month T3**		**T2-T3 ALL FCD**		**1 rd vs 2 rds**	
		1 rd. FCD N = 26	2 rds. FCD N = 145		1 rd. FCD N = 26	2 rds. FCD N = 145				
	All	Mean	Mean	All	Mean	Mean	Adj. diff.	P	Adj. diff.	P
Well-being	23.10	21.75	23.14	23.13	21.58	23.48	0.30	0.47	0.43	0.43
Self-efficacy	9.55	9.15	9.62	9.67	9.31	9.75	0.16	0.41	0.01	0.98
Self-worth	10.68	10.88	10.74	10.77	10.58	10.80	0.20	0.49	-0.15	0.76
Lack of parental satisfaction	0.62	1.15	0.49	0.66	1.08	0.60	0.10	0.48	-0.05	0.86
Parental stress	3.18	3.54	3.12	3.17	3.64	3.10	-0.04	0.83	-0.17	0.68
Family routines	42.73	43.88	42.37	42.63	42.84	42.55	-0.19	0.66	0.29	0.67
Supportive PB	26.77	25.44	26.83	26.35	25.08	26.46	-0.58	0.09	0.13	0.76
Hostile PB	12.99	13.28	13.33	13.01	13.60	12.80	0.06	0.84	-0.61	0.32
Network: Practical help	3.14	2.73	3.26	3.28	3.16	3.27	0.17	0.20	-0.36	**0.05**
Network: confidants	3.90	3.73	4.04	3.91	3.64	3.97	0.04	0.77	0.16	0.54
Network: loneliness	2.89	2.50	2.96	2.82	2.96	2.80	-0.09	0.45	-0.31	0.11
Parents: Conflicts with child	3.53	3.65	3.63	3.51	3.76	3.54	-0.05	0.78	-0.01	0.97
Parents: Conflicts with partner	1.81	2.31	1.72	1.93	3.15	1.78	0.17	0.49	-0.63	0.10
Parent: Participation in leisure activities	0.30	0.19	0.33	0.37	0.24	0.40	0.09	0.06	0.09	0.42
Feel comfortable in play with child	4.25	4.12	4.29	4.19	4.20	4.15	-0.08	0.27	-0.19	0.18
Need help to playtime with child	1.41	1.44	1.38	1.37	1.60	1.34	-0.05	0.44	-0.19	0.16
Initiates playtime with child	0.10	0.08	0.08	0.05	0.08	0.05	-0.04	0.20	-0.01	0.85
Parental screen time	4.89	4.35	4.92	4.90	4.68	4.92	-0.02	0.94	0.11	0.75
Learning activites in home	14.50	15.12	14.55	14.92	14.44	14.82	0.33	0.30	0.45	0.40
Frequency of family dinners	3.69	3.65	3.68	3.62	3.56	3.63	-0.08	0.17	0.01	0.95
Parents: Easiness of forming friendships	3.09	3.19	3.05	3.10	3.04	3.12	0.07	0.56	-0.00	1.00
Well-being (child age > 8 years)	44.34	42.07	45.15	46.49	45.21	47.04	1.98	0.31	-0.95	0.72
Well-being (child age < 8 years)	33.60	32.00	33.27	32.37	32.33	32.31	-1.09	0.09	0.06	0.98
Child: Conflicts with parents	2.88	2.80	2.97	2.88	3.20	2.85	-0.03	0.77	-0.39	**0.01**
Child: Conflicts with peers	2.52	2.32	2.57	2.55	2.60	2.55	0.04	0.61	-0.16	0.42

Child: Participation in leisure activities	0.48	0.52	0.50	0.56	0.52	0.59	0.14	**0.01**	0.12	0.19
Health of child	3.90	3.88	3.91	3.77	3.90	3.70	-0.20	0.23	-0.29	0.54
Child has sleep problems	2.05	2.12	2.04	1.94	1.88	1.94	-0.13	0.20	0.06	0.70
Child has one or more close friends	0.89	0.88	0.89	0.89	0.92	0.89	0.01	0.86	-0.04	0.54
Child screen time	3.72	3.80	3.68	3.64	3.92	3.57	-0.09	0.45	-0.28	0.16
Child: Easiness of forming friendships	3.68	3.80	3.74	3.73	3.52	3.72	0.05	0.60	0.34	**0.02**

Bold items are significant at $p < 0.05$; T2: Time 2 (after 6 months); T3: Time 3 (after 12 months); rd: rounds; WL: wait-list control; FCD: Family Club Denmark; adj. diff.: adjusted differencence.
